# Association of C1q/TNF-Related Protein-9 (CTRP9) Level with Obstructive Sleep Apnea in Patients with Coronary Artery Disease

**DOI:** 10.1155/2020/7281391

**Published:** 2020-08-05

**Authors:** Zexuan Li, Yunhui Du, Lixin Jia, Jingyao Fan, Ruifeng Guo, Xinliang Ma, Xiao Wang, Shaoping Nie, Yongxiang Wei

**Affiliations:** ^1^Emergency & Critical Care Center, Beijing Anzhen Hospital, Capital Medical University, Beijing, China; ^2^Beijing Institute of Heart, Lung and Blood Vessel Diseases, Beijing, China; ^3^Department of Cardiology, Beijing Anzhen Hospital, Capital Medical University, Beijing, China; ^4^Department of Emergency Medicine, Thomas Jefferson University, Philadelphia, PA, USA; ^5^Department of Otolaryngology Head & Neck Surgery, Beijing Anzhen Hospital, Capital Medical University, Beijing, China

## Abstract

**Background:**

Obstructive sleep apnea (OSA) is closely related to the incidence and progression of coronary artery disease (CAD), and the mechanisms linking OSA and CAD are multifactorial. C1q/TNF-related protein-9 (CTRP9) is a novel adipokine that protects the heart against ischemic injury and ameliorates cardiac remodeling. We aimed to ascertain the clinical relevance of CTRP9 with OSA prevalence in patients with CAD.

**Methods:**

From August 2016 to March 2019, consecutive eligible patients with CAD (*n* = 154; angina pectoris, *n* = 88; acute myocardial infarction [AMI], *n* = 66) underwent cardiorespiratory polygraphy. OSA was defined as an apnea-hypopnea index (AHI) ≥15 events·h^−1^. Plasma CTRP9 concentrations were measured by ELISA method.

**Results:**

Moderate/severe OSA was present in 89 patients (57.8%). CTRP9 levels were significantly decreased in the moderate/severe OSA group than in the no/mild OSA group (4.7 [4.1-5.2] ng/mL vs. 4.9 [4.4-6.0] ng/mL, *P* = 0.003). The difference between groups was only observed in patients with AMI (3.0 [2.3-4.9] vs. 4.5 [3.2-7.9], *P* = 0.009). Correlation analysis showed that CTRP9 levels were negatively correlated with AHI (*r* = −0.238, *P* = 0.003) and oxygen desaturation index (*r* = −0.234, *P* = 0.004) and positively correlated with left ventricular ejection fraction (*r* = 0.251, *P* = 0.004) in all subjects. Multivariate analysis showed that male gender (OR 3.099, 95% CI 1.029-9.330, *P* = 0.044), BMI (OR 1.148, 95% CI 1.040-1.268, *P* = 0.006), and CTRP9 levels (OR 0.726, 95% CI 0.592-0.890, *P* = 0.002) were independently associated with the prevalence of moderate/severe OSA.

**Conclusions:**

Plasma CTRP9 levels were independently related to the prevalence of moderate/severe OSA in patients with CAD, suggesting that CTRP9 might play a role in the pathogenesis of CAD exacerbated by OSA.

## 1. Background

Obstructive sleep apnea (OSA) is an increasingly recognized chronic disorder in adults [1, 2]. Recent evidence indicates OSA is closely related to the incidence and progression of coronary artery disease (CAD), and the prevalence of OSA is high (38% to 65%) in CAD patients [3, 4]. Prior reports and our study have shown that OSA was associated with an increased risk of recurrent cardiovascular events in patients with CAD and/or undergoing PCI [5–8]. However, the molecular mechanisms linking OSA and CAD are multifactorial.

OSA-mediated intermittent hypoxemia and sleep fragmentation triggers metabolic disorder, which is involved in cardiovascular impairment [9, 10]. Adiponectin is a cardioprotective adipokine, which have important role in insulin sensitivity, inflammation, and glucose homeostasis [11]. Previous studies have demonstrated that adiponectin levels were reduced in patients with severe OSA [12, 13]. Recently, a new family of secreted proteins, C1q tumor necrosis factor-related proteins (CTRPs), was found to have the same modular organization with adiponectin [14]. In all CTRPs families, C1q/TNF-related protein 9 (CTRP9) shares the highest degree (54%) of homology with adiponectin. CTRP9 levels are 100 times more than adiponectin in the myocardium [15]. Accumulating studies reveal that CTRP9 can protect the heart by attenuating atherosclerosis, alleviating acute ischemic injury, and attenuating adverse cardiac remodeling [16–18]. However, whether CTRP9 levels were altered by OSA in CAD patients remains undetermined. Therefore, we aimed to investigate the clinical relevance of CTRP9 with parameters of OSA, and whether CTRP9 is significantly associated with OSA prevalence in patients with CAD.

## 2. Methods

### 2.1. Study Design and Subjects

From August 2016 to March 2019, we consecutively enrolled patients aged 18 to 85 years with CAD (including angina pectoris (AP) and acute myocardial infarction (AMI)) and receiving overnight sleep study at the Emergency & Critical Care Center of Beijing Anzhen Hospital, Capital Medical University. AP included stable angina pectoris (SAP) and unstable angina pectoris. AMI included ST-segment elevation myocardial infarction and non-ST-segment elevation myocardial infarction. Among them, AMI is defined as the rise and/or fall of cardiac biomarker values such as CK-MB and/or troponin-T with at least one value above the 99th percentile upper reference limit and at least one of the following symptoms: ischemic, electrocardiogram changes indicative of new ischemia, development of pathologic Q waves, and imaging evidence of new loss of viable myocardium or new regional wall motion abnormality [19]. SAP's criteria include symptom of angina being stable for at least 6 months and having at least 50% luminal stenosis in at least one major coronary artery confirmed by coronary angiography. The criteria for unstable angina included symptoms of angina at rest, a new-onset exertional angina, or a recent acceleration of angina.

Exclusion criteria included cardiogenic shock, cardiac arrest, history of malignancy, hypertension (≥160/100 mmHg), diabetes, cancer, chronic kidney disease, valvular disease, stroke, thyroid disease, central sleep apnea, and patients without adequate and satisfactory sleep study or known use of continuous positive airway pressure (CPAP) treatment. Finally, one hundred and fifty-four CAD patients (88 AP and 66 AMI) were recruited. This study conformed to the Declaration of Helsinki. The Ethics Committee of Beijing Anzhen Hospital, Capital Medical University approved the study (2013025). All patients provided informed consent.

### 2.2. Overnight Sleep Study

All patients underwent an overnight sleep study after clinical stabilization during hospitalization (median 2 days (1 to 3 days) after admission) using a portable cardiorespiratory monitoring device (ApneaLink Air, Resmed, Australia). Nasal airflow, arterial oxygen saturation, thoracoabdominal movements, and snoring episodes were recorded. An apnea was defined by an absence of airflow lasting for ≥10 seconds. A hypopnea was defined as a reduction in airflow of >30% for ≥10 seconds and associated with a decrease in arterial oxygen saturation (SaO_2_) >4%. The apnea-hypopnea index (AHI) was defined as the number of apneas or hypopneas per hour of total recording time. Patients were divided into 2 groups: moderate/severe OSA group (AHI ≥15 events·h^−1^) and no/mild OSA group (AHI <15 events·h^−1^). All sleep studies were scored according to the American Academy of Sleep Medicine (AASM) 2007 guidelines. A minimum of 3 h of satisfactory signal recording was considered as a valid test. All studies were scored manually twice by independent sleep technologists (XW and JF) and reviewed by a senior consultant (YW) in cases of discrepancy.

### 2.3. Laboratory Measurements

All fasting venous blood samples were obtained the morning after the completion of overnight sleep study and overnight fast. We followed the manufacturer's recommendations. Blood samples were drawn into EDTA tubes and immediately centrifuged at 4°C, and plasma was frozen at -80°C for subsequent assays. Plasma glucose, cholesterol, triglycerides, high-sensitivity C-reactive protein, and homocysteine levels were analyzed using standard protocols of biochemistry laboratory. Plasma CTRP9 concentrations were determined with the sandwich method using a commercial ELISA kit (AVISCERA BIOSCIENCE, CA, USA; intra- and interassay CVs: 4-6% and 8-12%, respectively). We did not observe significant cross-reactivity or interference between human CTRP9 and analogs in our previous experiment. Samples were assayed in duplicate, and all results were reported as median.

### 2.4. Statistical Analysis

Continuous variables were presented as mean ± standard deviation (SD) or median (first and third quartiles) and were compared by Student's *t*-test or Mann–Whitney *U* test. Categorical variables were exhibited as the number (percentage) and were compared using chi-square test or Fisher's exact test. The correlations between plasma CTRP9 concentration and baseline and sleep parameters were determined by Spearman's correlation analysis. To identify independent factors of OSA incidence, binary logistic regression analysis was performed. Baseline variables that showed a univariate relationship with outcome were entered into the logistic regression models. All tests were 2-sided, and the value of *P* < 0.05 was considered statistically significant. Statistical analysis was performed with SPSS (version 25.0 IBM SPSS Inc, Armonk, NY).

## 3. Results

### 3.1. Baseline Characteristics of Subjects with Moderate/Severe OSA and No/Mild OSA

**Figure 1:**
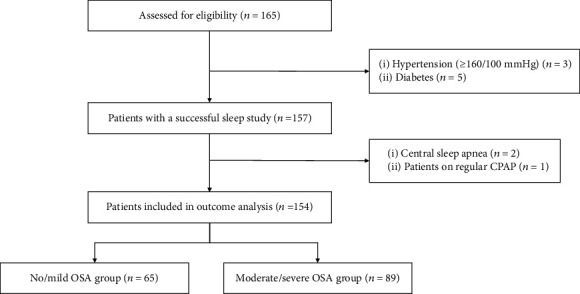
Study flowchart. CPAP: continuous positive airway pressure; OSA: obstructive sleep apnea.

In total, 165 consecutive eligible patients with CAD were prospectively enrolled, of whom 157 underwent a successful overnight sleep study. After exclusion of patients according to predefined criteria, 154 patients were included in the final analysis (Figure 1). Baseline characteristics of 154 CAD patients were listed in Table 1. Patients with moderate/severe OSA were more likely to be male and current smokers and had significantly higher body mass index (BMI), waist-to-hip ratio, and neck circumference compared with those with no/mild OSA. Other baseline information was generally well matched between moderate/severe OSA group and no/mild OSA groups.

### 3.2. Sleep Study Results

Based on the criteria of AHI ≥15, the prevalence of moderate/severe OSA was 57.8% in this CAD cohort. Patients with moderate/severe OSA exhibited higher AHI and oxygen desaturation index (ODI), lower minimum and average oxygen saturation, and more time of SaO2 <90% compared with those with no/mild OSA (Table 1).

### 3.3. Plasma CTRP9 Concentrations in Subjects with Moderate/Severe OSA and No/Mild OSA

**Figure 2:**
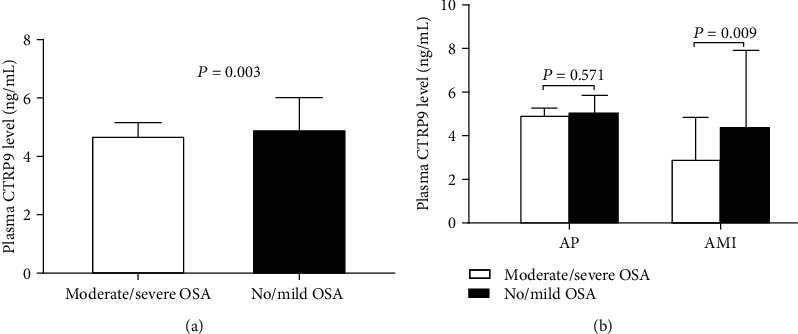
Plasma CTRP9 levels between moderate/severe OSA and no/mild OSA groups in patients with CAD (a). Plasma CTRP9 levels between moderate/severe OSA and no/mild OSA groups in AP and AMI subgroups (b). AMI: acute myocardial infarction; AP: angina pectoris; CTRP9: C1q/TNF-related protein 9; OSA: obstructive sleep apnea.

Plasma CTRP9 concentrations were significantly decreased in the moderate/severe OSA group than in the no/mild OSA group (4.7 [4.1-5.2] ng/mL vs. 4.9 [4.4-6.0] ng/mL, *P* = 0.003) (Table 1 and Figure 2(a)). When we stratified the CAD patients into AP and AMI subgroup, the plasma CTRP9 levels were significantly lower in the moderate/severe OSA group only in patients with AMI (3.0 [2.3-4.9] ng/mL vs. 4.5 [3.2-7.9] ng/mL, *P* = 0.009), but not in patients with AP (5.0 [4.7-5.3] ng/mL vs. 5.1 [4.7-5.9] ng/mL, *P* = 0.571) (Figure 2(b)).

### 3.4. Correlation of CTRP9 Levels with Sleep Parameters and LVEF

**Figure 3:**
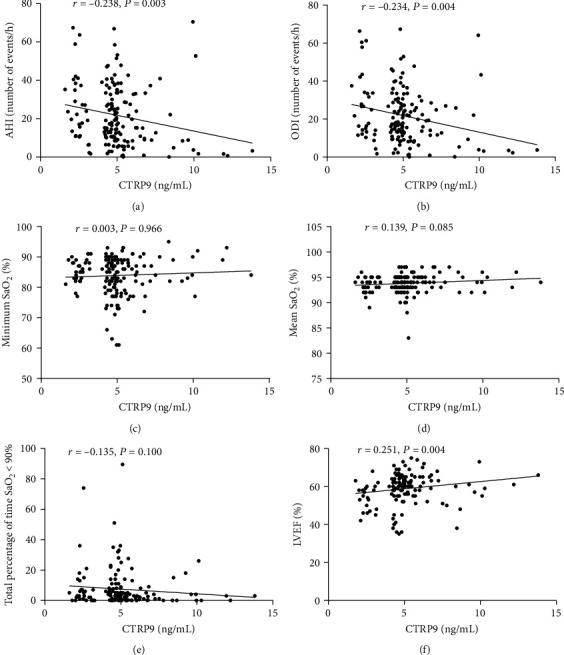
Correlation between CTRP9 and parameters of sleep study (a–e) and LVEF (%) (f). AHI: apnea-hypopnea index; LVEF: left ventricular ejection fraction; ODI: oxygen desaturation index; SaO_2_: arterial oxygen saturation.

**Figure 4:**
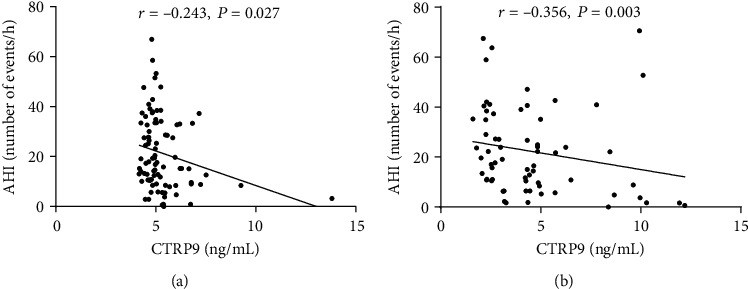
Correlation between CTRP9 and AHI in patients with AP (a) and AMI (b). AHI: apnea-hypopnea index; AMI: acute myocardial infarction; AP: angina pectoris.

Scatter plot showed the correlation of CTRP9 levels with sleep parameters and LVEF (Figure 3). CTRP9 levels were negatively correlated with AHI (*r* = −0.238, *P* = 0.003) and oxygen desaturation index (*r* = −0.234, *P* = 0.004) and positively correlated with left ventricular ejection fraction (LVEF) (*r* = 0.251, *P* = 0.004) in all subjects. On the other hand, CTRP9 concentrations had no significant correlation with minimum SaO_2_, mean SaO_2_, and the time of SaO_2_ <90%. In subgroup patients with AP and AMI, there was also a significant correlation between CTRP9 and AHI (Figure 4).

### 3.5. Baseline Characteristics according to Tertiles of CTRP9 Levels

All subjects were categorized into trisection according to CTRP9 tertiles (T1: <4.47 ng/mL; T2: 4.47-5.07 ng/mL; T3: >5.07 ng/mL). The baseline characteristics of each category are shown in Table S1. There were significant differences among the tertiles in terms of systolic and diastolic blood pressure, LVEF, AHI, and ODI.

### 3.6. Plasma CTRP9 and the Prevalence of OSA

To evaluate the association between CTRP9 and OSA, univariate and multivariate logistic regression analyses were performed. In univariate logistic regression, we found that gender, BMI, neck circumference, HDL-cholesterol, and CTRP9 levels were significantly associated with the prevalence of OSA. In the multivariate model, only male gender (OR 3.099, 95% CI 1.029-9.330, *P* = 0.044), BMI (OR 1.148, 95% CI 1.040-1.268, *P* = 0.006), and CTRP9 levels (OR 0.726, 95% CI 0.592-0.890, *P* = 0.002) were independently associated with the prevalence of OSA (Table 2). Neck circumference did not enter into the multivariate regression due to its multicollinearity.

To further explore the risk factors for OSA comorbidity in patients with CAD, we established multiple regression models based on the CTRP9 tertile. In model 1 without adjustment, subjects with low and moderate CTRP9 levels had significantly higher risk of OSA compared with those with high levels. This trend was further intensified after adjustment for age, gender, and BMI (Table S2).

### 3.7. Correlation between AHI and Other Variables

To evaluate the association between CTRP9 and AHI, correlation analysis and multivariate linear regression analysis were performed (Table S3). Spearman's correlation analysis illustrated that AHI was positively correlated with BMI (*r* = 0.256, *P* = 0.001), waist-to-hip ratio (*r* = 0.184, *P* = 0.024), and neck circumference (*r* = 0.347, *P* = 0.000) and negatively correlated with CTRP9 levels (*r* = −0.238, *P* = 0.003) in all subjects. In the multivariate linear regression model, CTRP9 levels, sex, and BMI were independent factors associated with AHI (*P* < 0.05, respectively). Waist-to-hip ratio and neck circumference were excluded from the model due to their high multicollinearity with BMI.

## 4. Discussion

In the present study, we first demonstrated the clinical relevance of CTRP9 with OSA in patients with CAD. The plasma CTRP9 levels were significantly decreased in moderate/severe OSA versus no/mild OSA groups, which was driven by the difference in patients with AMI. Lower CTRP9 level was an independent factor related to OSA prevalence even after adjusting for other confounding factors.

Emerging evidence has demonstrated a close relationship between OSA and CAD [4, 5, 20]. Also, prior reports have shown that OSA-mediated chronic intermittent hypoxia (CIH), triggered by repetitive episodes of apneas and hypopneas, exacerbates metabolic dysfunction including insulin resistance and nonalcoholic fatty liver disease [10, 21]. Mechanistically, recurrent cycles of hypoxemia with reoxygenation promote oxidative stress, systemic inflammation, and endothelial dysfunction, all contributing to the pathogenesis of diabetes [22]. Our recent study indicated that OSA was associated with increased risk of 1-year cardiovascular events following acute coronary syndrome only in patients with diabetes or poor glucose control, but not in patients without diabetes [23]. Therefore, metabolic disorders may be involved in OSA-induced incidence and aggravation of CAD.

Findings from animal models and patients have demonstrated that the levels of adiponectin, a classical cardioprotective adipokine, were decreased under CIH/OSA [12, 13, 24]. However, the results were distinct and CPAP intervention did not increase adiponectin levels [25]. C1q tumor necrosis factor-related proteins (CTRPs) is a highly conserved family of adiponectin paralogs, which includes fifteen family members [14]. Among them, CTRP9 shares the greatest homology with adiponectin and is highly expressed in the adult heart. Besides the metabolic regulatory properties, CTRP9 had important local cardiac biological function [15]. CTRP9 supplementation attenuated cardiac remodeling and improved contractile function postmyocardial infarction (MI) [17]. Moreover, clinical studies have shown decreased CTRP9 levels in patients with MI as well as heart failure with reduced ejection fraction [26, 27]. In the present study, we found that in patients with CAD, the CTRP9 levels were further reduced in moderate/severe OSA group compared with no/mild OSA group, and the CTRP9 concentrations were inversely correlated to the AHI levels. The difference between groups was only observed in patients with AMI, because in such circumstances, the heart may be in a more vulnerable state sensitive to the negative consequences of OSA [4]. Also, our recent study found that cardiac CTRP9 gene and protein levels were significantly reduced in CIH+MI animals [28]. These findings indicate that CTRP9 may be involved in the pathogenesis of CAD and related complications exacerbated by OSA.

Obesity is a major risk factor for OSA, because it contributes significantly to pharyngeal airway narrowing [10]. Our study also showed that BMI level significantly predicted higher risk of OSA. Noteworthy, multivariate regression analysis showed that CTRP9 levels were independently associated with the prevalence of OSA. Furthermore, the results from basic study demonstrated that CTRP9 supplementation significantly attenuated CIH-exacerbated post-MI remodeling and improved cardiac function [28]. These findings support CTRP9 as a potential therapeutic target by restoring cardiac function in CAD patients with OSA.

### 4.1. Limitations

First, due to the cross-sectional design, the causal relationship could not be confirmed. Second, this is a single-center study that recruited only East-Asian patients. Studies pertaining to other ethnicities are needed. Third, the plasma levels of other members of CTRP family need to be examined in future studies.

## 5. Conclusions

In conclusion, the present study demonstrated that CTRP9 levels were significantly reduced in moderate/severe OSA versus no/mild OSA groups in patients with CAD. Lower CTRP9 levels were independently associated with OSA prevalence after adjusting for traditional contributing factors. These results support the role of CTRP9 in linking OSA and pathogenesis of CAD.

## Figures and Tables

**Table 1:** Baseline characteristics of subjects with moderate/severe OSA and no/mild OSA.

Variables	All (*n* = 154)	Moderate/severe OSA (*n* = 89)	No/mild OSA (*n* = 65)	*P*
Age (years)	54.9 ± 9.4	54.4 ± 8.8	55.1 ± 10.3	0.619
Male (%)	136 (88.3)	83 (93.3)	53 (81.5)	0.025
BMI (kg/m^2^)	27.2 ± 3.7	27.9 ± 3.7	26.4 ± 3.9	0.012
Waist-to-hip ratio	0.97 (0.94-1.01)	0.98 (0.95-1.02)	0.97 (0.93-1.00)	0.032
Neck circumference (cm)	40.0 (38-42)	40.5 (38-42)	40.0 (37-44)	<0.001
Systolic BP (mm/Hg)	126 (116-139)	129 (115-140)	125 (115-136)	0.389
Diastolic BP (mm/Hg)	76 (70-86)	79 (70-87)	74 (70-85)	0.176
Hypertension (%)	89 (57.8)	54 (60.7)	35 (53.8)	0.397
Hyperlipidemia (%)	38 (24.7)	23 (25.8)	15 (23.1)	0.694
Current smoking (%)	76 (49.4)	53 (59.6)	23 (35.4)	0.015
Previous CAD (%)	49 (31.8)	29 (32.6)	20 (30.8)	0.811
Previous myocardial infarction (%)	19 (12.3)	12 (13.5)	7 (10.8)	0.613
Previous PCI (%)	24 (15.6)	18 (20.2)	6 (9.2)	0.063
LDL-cholesterol (mmol/L)	2.3 (1.8-3.1)	2.4 (1.9-3.0)	2.1 (1.7-3.0)	0.635
HDL-cholesterol (mmol/L)	1.03 (0.89-1.18)	1.03 (0.91-1.20)	1.01 (0.89-1.13)	0.010
Total cholesterol (mmol/L)	3.9 (3.3-5.0)	4.2 (3.45-4.9)	3.8 (3.2-4.8)	0.948
Triglyceride (mmol/L)	1.4 (1.0-2.3)	1.5 (1.0-2.2)	1.3 (0.9-2.1)	0.448
LVEF (%)	60 (55-65)	62 (60-65)	55 (48-60)	0.582
CTRP9 (ng/mL)	4.9 (4.3-5.5)	4.7 (4.1-5.2)	4.9 (4.4-6.0)	0.003
hsCRP (mg/L)	1.5 (0.6-5.1)	1.2 (0.5-3.3)	2.0 (0.6-5.9)	0.103
HCY (*μ*mol/L)	12.7 (9.4-19.1)	12.7 (9.3-16.0)	11.9 (9.5-19.2)	0.618
HbA1c (%)	5.8 (5.4-6.0)	5.8 (5.5-6.2)	5.6 (5.4-6.0)	0.293
Fasting glucose (mmol/L)	5.48 (5.07-5.95)	5.54 (5.07-6.00)	5.48 (5.12-5.86)	0.220
AHI (events/h)	19.1 (9.9-33.5)	29.0 (21.9-38.8)	8.5 (4.3-10.9)	<0.001
ODI (events/h)	20.7 (11.7-30.7)	27.2 (21.2-36.2)	10.5 (4.6-13.9)	<0.001
Minimum SaO_2_ (%)	85 (81-89)	83 (79-87)	88 (83-90)	<0.001
Mean SaO_2_ (%)	94 (93-95)	93 (93-95)	94 (94-95)	0.001
Time with SaO_2_ <90% (%)	3.0 (0.2-8.0)	5.0 (2.0-14.0)	0.8 (0-3.3)	<0.001

Data are presented as mean ± SD: median (first quartile: third quartile): or **n** (%). AHI: apnea-hypopnea index; BMI: body mass index; BP: blood pressure; CAD: coronary artery disease; CTRP9: C1q/TNF-related protein 9; HbA1c: glycated hemoglobin; HCY: homocysteine; HDL: high-density lipoprotein; hsCRP: high-sensitivity C-reactive protein; LDL: low-density lipoprotein; LVEF: left ventricular ejection fraction; ODI: oxygen desaturation index; OSA obstructive sleep apnea; PCI: percutaneous coronary intervention; SaO_2_: arterial oxygen saturation.

**Table 2:** Independent factors associated with OSA in binary logistic regression models.

Variables	Univariate			Multivariate		
	*β* ± SE	OR (95% CI)	*P*	*β* ± SE	OR (95% CI)	*P*
Age	−0.008 ± 0.017	0.992 (0.959-1.026)	0.630			
Male	1.142 ± 0.530	3.132 (1.108-8.851)	0.031	1.131 ± 0.562	3.099 (1.029-9.330)	0.044
BMI	0.113 ± 0.046	1.119 (1.023-1.225)	0.015	0.138 ± 0.051	1.148 (1.040-1.268)	0.006
Waist-to-hip ratio	0.979 ± 1.908	2.663 (0.063-112.055)	0.608			
Neck circumference	0.186 ± 0.055	1.205 (1.082-1.341)	0.001			
Systolic BP	0.004 ± 0.010	1.004 (0.985-1.023)	0.693			
Diastolic BP	0.018 ± 0.014	1.018 (0.991-1.047)	0.197			
Hypertension	0.279 ± 0.330	1.322 (0.692-2.526)	0.414			
Hyperlipidemia	0.150 ± 0.381	1.162 (0.550-2.452)	0.694			
Current smoking	0.835 ± 0.465	2.304 (0.926-5.732)	0.073			
Previous CAD	0.084 ± 0.351	1.087 (0.546-2.165)	0.811			
Previous myocardial infarction	0.256 ± 0.506	1.291 (0.479-3.484)	0.614			
Previous PCI	0.913 ± 0.503	2.493 (0.930-6.685)	0.069			
LDL-cholesterol	−0.060 ± 0.162	0.942 (0.686-1.294)	0.713			
HDL-cholesterol	−1.602 ± 0.792	0.202 (0.043-0.951)	0.043	−1.220 ± 0.851	0.295 (0.056-1.565)	0.152
Total cholesterol	−0.064 ± 0.140	0.938 (0.712-1.235)	0.647			
Triglyceride	0.088 ± 0.169	1.092 (0.784-1.520)	0.603			
LVEF	0.003 ± 0.021	1.003 (0.962-1.046)	0.871			
CTRP9	−0.256 ± 0.093	0.774 (0.645-0.929)	0.006	−0.320 ± 0.104	0.726 (0.592-0.890)	0.002
hsCRP	0.024 ± 0.026	1.024 (0.973-1.077)	0.357			
HCY	0.010 ± 0.016	1.010 (0.979-1.041)	0.532			
HbA_1_C	0.525 ± 0.344	1.690 (0.861-3.318)	0.127			
Fasting glucose	0.406 ± 0.208	1.501 (0.999-2.254)	0.051			

BMI: body mass index; BP: blood pressure; CAD: coronary artery disease; CTRP9: C1q/TNF-related protein 9; HbA1c: glycated hemoglobin; HCY: homocysteine; HDL: high-density lipoprotein; hsCRP: high-sensitivity C-reactive protein; LDL: low-density lipoprotein; LVEF: left ventricular ejection fraction; PCI: percutaneous coronary intervention.

## Data Availability

Data generated or analyzed during this study are included in this published article.

## Abbreviations

AASM: American Academy of Sleep Medicine
AHI: Apnea-hypopnea index
AMI: Acute myocardial infarction
AP: Angina pectoris
BMI: Body mass index
BP: Blood pressure
CAD: Coronary artery disease
CI: Confidence interval
CPAP: Continuous positive airway pressure
CTRP9: C1q/TNF-related protein 9
ELISA: Enzyme-linked immunosorbent assay
HbA1c: Glycated hemoglobin
HCY: Homocysteine
HDL: High-density lipoprotein
hsCRP: High-sensitivity C-reactive protein
LDL: Low-density lipoprotein
LVEF: Left ventricular ejection fraction
ODI: Oxygen desaturation index
OR: Odds ratio
OSA: Obstructive sleep apnea
PCI: Percutaneous coronary intervention
SaO_2_: Arterial oxygen saturation
SD: Standard deviation.

## Supplementary Materials

Supplementary Materials Table S1: baseline characteristics according to tertiles of CTRP9 levels. Table S2: OSA risk according to CTRP9 tertiles in multivariable logistic regression. Table S3: correlation between AHI and other variables.
