# Supplementary material for: Association of C1q/TNF-Related Protein-9 (CTRP9) Level with Obstructive Sleep Apnea in Patients with Coronary Artery Disease
Source: Mediators Inflamm. 2020 Aug 5;2020:7281391. doi: 10.1155/2020/7281391 (PMC7426787; doi:10.1155/2020/7281391)
Supplement: Supplementary Materials — Table S1: baseline characteristics according to tertiles of CTRP9 levels. Table S2: OSA risk according to CTRP9 tertiles in multivariable logistic regression. Table S3: correlation between AHI and other variables. [file 7281391.f1.docx]

**Table S1. Baseline characteristics according to** **tertiles of CTRP9 levels (T1: < 4.47ng/mL: T2: 4.47-5.07ng/mL: T3: > 5.07ng/mL)**

| **Variables** | **T1 (n = 51)** | **T2 (n = 52)** | **T3 (n = 51)** | ***P*** |
| --- | --- | --- | --- | --- |
| Age (years) | 55.4 ± 9.5 | 54.5 ± 9.5 | 54.4 ± 9.6 | 0.836 |
| Male (%) | 47 (92.2) | 46 (88.5) | 43 (84.3) | 0.467 |
| BMI (kg/m^2^) | 26.6 ± 3.8 | 27.3 ± 4.0 | 28.1 ± 3.7 | 0.158 |
| Waist-to-hip ratio | 0.97 (0.94-1.00) | 0.96 (0.94-1.01) | 0.99 (0.96-1.02) | 0.135 |
| Neck circumference (cm) | 40 (37-44) | 40 (38-41) | 40 (38-42) | 0.906 |
| Systolic BP (mm/Hg) | 121 (111-135) | 130 (120-144) | 130 (118-140) | 0.025 |
| Diastolic BP (mm/Hg) | 74 (66-80) | 82 (70-91) | 76 (70-85) | 0.040 |
| Hypertension (%) | 26 (51.0) | 28 (53.8) | 35 (68.6) | 0.153 |
| Hyperlipidemia (%) | 9 (17.6) | 14 (26.9) | 15 (29.4) | 0.348 |
| Current smoking (%) | 29 (56.9) | 26 (50) | 21 (41.2) | 0.384 |
| Previous CAD (%) | 13 (25.5) | 20 (38.5) | 16 (31.4) | 0.367 |
| Previous myocardial infarction (%) | 3 (5.89) | 8 (15.4) | 8 (15.7) | 0.230 |
| Previous PCI (%) | 6 (11.8) | 9 (17.3) | 9 (17.6) | 0.654 |
| LDL-cholesterol (mmol/L) | 2.2 (1.8-2.9) | 2.4 (1.9-3.0) | 2.1 (1.7-3.0) | 0.382 |
| HDL-cholesterol (mmol/L) | 1.00 (0.88-1.15) | 1.03 (0.91-1.22) | 1.03 (0.90-1.18) | 0.620 |
| Total cholesterol (mmol/L) | 3.78 (3.26-4.67) | 4.26 (3.45-4.91) | 3.82 (3.26-5.02) | 0.295 |
| Triglyceride (mmol/L) | 1.17 (0.84-1.98) | 1.48 (0.93-2.13) | 1.66 (1.09-2.30) | 0.062 |
| LVEF (%) | 58 (48-61) | 62 (58-66) | 61 (57-65) | 0.001 |
| hsCRP (mg/L) | 1.8 (0.5-6.1) | 1.3 (0.6-4.1) | 1.1 (0.5-3.9) | 0.685 |
| HCY (μmol/L) | 11.7 (9.8-19.2) | 13.3 (9.2-18.3) | 11.9 (9.0-15.9) | 0.669 |
| HbA1C (%) | 5.7 (5.4-6.0) | 5.8 (5.5-6.2) | 5.7 (5.4-5.9) | 0.329 |
| Fasting glucose (mmol/L) | 5.5 (5.1-5.9) | 5.7 (5.2-6.1) | 5.5 (5.1-5.9) | 0.251 |
| AHI (events/h) | 19.1 (11.1-37.3) | 22.7 (12.5-34.5) | 10.8 (4.8-28.5) | 0.004 |
| ODI (events/h) | 20.7 (13.2-34.0) | 19.6 (13.3-30.5) | 14.7 (6.0-25.1) | 0.010 |
| Minimum SaO_2_ (%) | 86 (82-89) | 83 (80-88) | 86 (81-89) | 0.140 |
| Mean SaO_2_ (%) | 94 (93-95) | 94 (93-95) | 94 (93-95) | 0.223 |
| Time with SaO_2_ < 90% (%) | 5.0 (0.3-8.8) | 3.0 (0.3-11.0) | 2.4 (0.0-5.0) | 0.261 |

Data are presented as mean ± SD: median (first quartile: third quartile): or n (%). AHI indicates apnea-hypopnea index; BMI: body mass index; BP: blood pressure; CAD: coronary artery disease; CTRP9: C1q/TNF-related protein 9; HbA1c: glycated hemoglobin; HCY: homocysteine; HDL: high-density lipoprotein; hsCRP: high-sensitivity C-reactive protein; LDL: low-density lipoprotein; LVEF: left ventricular ejection fraction; ODI: oxygen desaturation index; OSA obstructive sleep apnea; PCI: percutaneous coronary intervention; SaO_2_: arterial oxygen saturation.

**Table S2. OSA risk according to CTRP9 tertiles in multivariable logistic regression**

|  | OR (95%CI) | | |
| --- | --- | --- | --- |
|  | T1 | T2 | T3 (Reference) |
| Model 1 | 2.220 (1.004-4.908) | 2.714 (1.217-6.050) | 1 |
| *P* value | 0.049 | 0.015 |  |
| Model 2 | 2.096 (0.935-4.698) | 2.684 (1.188-6.063) | 1 |
| *P* value | 0.072 | 0.018 |  |
| Model 3 | 2.714 (1.147-6.421) | 3.254 (1.383-7.658) | 1 |
| *P* value | 0.023 | 0.007 |  |

Model 1: unadjusted.

Model 2: adjusted for age and sex.

Model 3: adjusted for age: sex and BMI.

BMI indicates body mass index.

**Table S3.** **Correlation between AHI and other variables**

| **Variables** | **All subjects (n=154)** | | **Multivariate Linear regression analysis** | |
| --- | --- | --- | --- | --- |
|  | ***r*** | **P** | **Standard β** | ***P*** |
| Age | 0.005 | 0.947 | 0.042 | 0.597 |
| Male | - | - | -0.159 | 0.041 |
| BMI | 0.256 | 0.001 | 0.295 | 0.000 |
| Waist-to-hip ratio | 0.184 | 0.024 | - | - |
| Neck circumference | 0.347 | 0.000 | - | - |
| Systolic BP | 0.078 | 0.334 | - | - |
| Diastolic BP | 0.110 | 0.173 | - | - |
| LDL-cholesterol | 0.053 | 0.514 | - | - |
| HDL-cholesterol | -0.140 | 0.084 | - | - |
| Total Cholesterol | 0.010 | 0.898 | - | - |
| Triglyceride | -0.018 | 0.828 | - | - |
| LVEF | -0.017 | 0.848 | - | - |
| hsCRP | 0.133 | 0.101 | - | - |
| HCY | 0.110 | 0.191 | - | - |
| HbA1C | 0.148 | 0.079 | - | - |
| Fasting glucose | 0.121 | 0.134 | - | - |
| CTRP9 | -0.238 | 0.003 | -0.229 | 0.003 |

BMI indicates body mass index; BP: blood pressure; HbA1c: glycated hemoglobin; HCY: homocysteine; HDL: high-density lipoprotein; hsCRP: high-sensitivity C-reactive protein; LDL: low-density lipoprotein; LVEF: left ventricular ejection fraction.
